# Anti-IL6 treatment of serious COVID-19 disease: A monocentric retrospective experience

**DOI:** 10.1097/MD.0000000000023582

**Published:** 2021-01-08

**Authors:** Laura Castelnovo, Antonio Tamburello, Alfredo Lurati, Eleonora Zaccara, Maria Grazia Marrazza, Micol Olivetti, Nicola Mumoli, Daniela Mastroiacovo, Daniele Colombo, Elisabetta Ricchiuti, Paolo Vigano’, Faggioli Paola, Antonino Mazzone

**Affiliations:** a Department of Internal Medicine, ASST Ovest Milanese Ospedale di Legnano; b Department of Rheumatology; c Department of Internal Medicine, ASST Ovest Milanese Ospedale di Magenta; d Department of Infectious Diseases, ASST Ovest Milanese Ospedale di Legnano, Italy.

**Keywords:** coronavirus, COVID-19, cytokine, interleukin-6, tocilizumab

## Abstract

COVID-19 is causing a high influx of patients suffering from serious respiratory complications leading the necessity to find effective therapies. These patients seem to present with cytokine perturbation and high levels of IL6. Tocilizumab and sarilumab could be effective in this condition.

We retrospectively collected data about 112 consecutive hospitalized in a single center.

Fifty (IL6 group) treated with tocilizumab (8 mg/kg intravenously [IV], 2 infusions 12 hours apart) or sarilumab 400 mg IV once and 62 treated with the standard of care but not anti-cytokine drugs (CONTROL group).

To determine whether anti-IL6 drugs are effective in improving prognosis and reducing hospitalization times and mortality in COVID-19 pneumonia.

To date 84% (42/50) of IL6 group patients have already been discharged and only 2/50 are still recovered and intubated in intensive care. Six/fifty patients (12%) died: 5/6 due to severe respiratory failure within a framework of severe acute respiratory distress syndrome (ARDS), 1 suffered an acute myocardial infarction, and 1 died of massive pulmonary thromboembolism. There were no adverse treatment events or infectious complications. Compared to the CONTROL group they showed a lower mortality rate (12% versus 43%), for the same number of complications and days of hospitalization.

Anti-IL6 drugs seem to be effective in the treatment of medium to severe forms of COVID-19 pneumonia reducing the risk of mortality due to multi-organ failure, acting at the systemic level and reducing inflammation levels and therefore microvascular complications. However, it is essential to identify the best time for treatment, which, if delayed, is rendered useless as well as counterproductive. Further studies and ongoing clinical trials will help us to better define patients eligible as candidates for more aggressive intervention.

**Key Points**

- What is already known about this subject? Anti-IL6 drugs tocilizumab and sarilumab are known to be very effective in the treatment of rheumatoid arthritis, juvenile idiopathic arthritis, systemic, giant cell arteritis, and Cytokine Release Syndrome which occurs during therapy with Car-T. Recent studies have hypothesized their effectiveness also in the treatment of COVID pneumonia 19.
- What does this study add? This work to our knowledge it is one of the most numerous cases described to date. Fifty consecutive patients affected by COVID-19 pneumonia were treated with anti-IL6 drugs between March and May 2020. Findings: 84% of IL6 group patients have already been discharged, 4% are still hospitalized but 6/50 (12%) patients died. There were no adverse treatment events or severe infectious complications. Compared to the CONTROL group they showed a lower mortality rate for the same number of complications and days of hospitalization.
- How might this impact on clinical practice? The results could help identify the best therapeutic approach of an emerging pathology, currently burdened by high hospitalization rate and mortality. Anti-IL6 drugs could help the physician to avoid complications and reduce the mortality rate in COVID-19 infection, but it is essential to identify the best time for treatment, which, if delayed, is rendered useless as well as counterproductive. Much remains to be understood for better characterizing the COVID-19 disease and the findings so far should be evaluated systematically in larger patient cohorts to allow reliable conclusions.

## 1 Introduction

COVID-19 (WHO denomination) is a novel pneumonia caused by SARS-CoV-2 coronavirus (acronym for severe acute respiratory syndrome coronavirus 2), which emerged in Wuhan, in the province of Hubei, China in December 2019. COVID-19 broke-out aggressively in January 2020 following the human flow from Wuhan to other cities until it has been declared a pandemic. The region of Lombardy has the highest number of cases of COVID-19 in Italy and appears to be the epicenter of the Italian outbreak causing a serious emergency situation in all local health facilities as a vast group of patients with COVID-19 disease were critical. The clinical features of COVID-19 are similar to SARS and MERS, with typical manifestation of pneumonia and acute respiratory infection symptoms that can quickly degenerate into respiratory failure. So, the pandemic leaded to the need to find effective and fast therapeutic strategies. Some studies have just shown that patients requiring intensive care develop a cytokine storm that induces extensive lung damage. This situation is manifested in high levels of serum interleukin (IL)-2R, IL6, IL10, and TNF-α and absolute numbers of CD4+ and CD8+ T lymphocytes low. Starting from this data tocilizumab, a humanized monoclonal antibody against the interleukin-6 receptor (IL-6R) approved for the treatment of rheumatoid arthritis, juvenile idiopathic arthritis,^[1,2]^ systemic, giant cell arteritis,^[3]^ and Cytokine Release Syndrome (CRS) occurring during therapy with Car-T,^[4,5]^ has been proposed as a potentially effective drug in severe forms of COVID-19 pneumonia.^[6]^ Similarly, sarilumab is a human monoclonal which binds specifically to IL6 (IL-6Rα) receptors both soluble and bound to the membrane and inhibits IL6 mediated signaling. It is approved for the treatment of rheumatoid arthritis.^[11]^ Based on the existing literature, it has also been proposed as a potentially effective drug in the treatment of lung complications due to COVID-19.^[10,12]^ A great number of international trials regarding the use of tocilizumab and sarilumab in severe pneumonia due to COVID-19^[13]^ are ongoing.

## 2 Methods

### 2.1 Patients

Patients hospitalized for COVID-19 from March 6 to May 30 2020 were retrospectively enrolled in our observational study. All patients gave written informed consent to off-label use of tocilizumab.

Due to the emergency situation, it was not possible to conduct a randomized controlled trial. We divided them in 2 groups: IL6-group and CONTROL group depending on the type of treatment received.

### 2.2 Treatments

IL6-group included patients treated with tocilizumab 8 mg/kg intravenously (IV) (max usable dosage 800 mg IV) in a Saline Solution 100 mL in 60 minutes. A second infusion was performed after 12 hours. 24 hours after this infusion, a third infusion was indicated in case of non-improvement of the parameters. Two patients of this group were treated with sarilumab 400 mg IV in a Saline Solution 100 mL in 60 minutes; 12 hours after this infusion, a possible second infusion of sarilumab was indicated in case of non-improvement of the clinical and respiratory parameters.^[8]^ These treatments were not contraindicated in association with antiviral therapy (Remdesivir or Ritonavir/Lopinavir) and/or antimalarials (chloroquine o Oh-chloroquine) and low molecular weight heparin (LMWH), according to current guidelines.^[9]^ Patients included in CONTROL group received the standard of care (SOC) of the treatment, according to the indications of the national guidelines: antiviral, antibiotics, antimalarial, LMHW, and steroids when required.

### 2.3 Eligibility criteria

Hospitalized patients with confirmed COVID-19 were included in IL6 group if they fulfilled primary criteria: age > 18 years, presence of severe interstitial pneumonia on chest CT or X-ray with respiratory failure requiring C-PAP with FI02 > 40% and/or Venturi Mask > 50%, high levels of d-dimer (>1500 ng/mL), or d-dimer in progressive rise and/or hyperferritinemia (>500 ng/mL). Patients with increase in AST/ALT > 5 times normal levels and/or neutrophils < 500 cell/mmc and/or platelets < 50,000 cell/mmc, documented sepsis from pathogens other than COVID-19 and/or infection in progress (e.g., dermohypoderma not controlled by antibiotic therapy) or high levels of procalcitonin, personal history of complicated diverticulitis or intestinal perforation, pregnancy, immunosuppressive anti-rejection therapy and/or therapy with dabigatran were not considered suitable for treatment. Patients that not fulfilled inclusion criteria (CONTROL group) were treated with SOC.

### 2.4 Outcomes

Clinical features such as body temperature and oxygen saturations were recorded daily. A white blood cell count, C reactive protein (CPR), PcT, d-dimer, creatinin, and transaminases were performed repeatedly (time 0–24 and 36). All patients received a chest X-ray or a CT scan on admission and prior to hospital discharge. Samples of serum were stocked and frozen, for a future dosage of the IL6 levels (immediately before the monoclonal antibodies infusion and after 24–36 hours). Patients’ clinical status was assessed categorizing them discharged, still hospitalizated or died. Overall survival and the proportion of patients with clinical improvement were assessed. Predictors of mortality and clinical improvement were analyzed in tocilizumab patients with specific attention to infectious or thromboembolic complications.

### 2.5 Statistical analysis

Descriptive statistics summarized data using the mean and median, as appropriate based on the variable distribution, or frequency rates and percentages for dichotomous variables. Comparisons between IL6 and CONTROL groups were made by parametric tests or no parametric tests for continuous variables. Bivariate correlation was made by Spearman tests. All statistical analyzes were performed using SPSS version 13.0 software (SPSS Inc).

### 2.6 Patient and public involvement

Patients were not involved in the design of this study as it involved only observational analysis of an anonymized, pre-existing, routinely collected dataset. Written consent was obtained from all patients; they received information about their clinical status and about the treatment during care and signed an informed consent for the use of the off-label monoclonal antibodies (648/96 Law). The study was conducted in accordance with the ethical principles of the Helsinki Declaration.

## 3 Results

Between March 6 and May 7 2020, a total of 112 consecutive patients (34 F and 77 M), mean age 65 years (median 65, range 29–21), were admitted to our hospital due to COVID-19 pneumonia; oxygen saturation equal or below 95% was documented in all of them. The most frequent registered comorbidity was arterial hypertension (45/112 patients, 40%) followed by diabetes (23/112, 20%) but 57/112 (50.8%) patients had not taken any therapy before hospital admission and nor did they have any significant medical history. Antimalarials (hydroxychloroquine minimum dose 200 mg/bid for 7 days), LMWH, steroids (methylprednisolone or prednisolone), and antibiotics were used in all patients; only a small part was treated with antivirals (mostly Lopinavir/Ritonavir 400/100 mg bid). Main characteristics of the enrolled patients, divided by the 2 groups, are summarized in Table 1. Basal characteristics of the patients in the 2 groups are similar, the most inconsistent figure is the need for ventilatory support with Continuous Positive Airway Pressure (CPAP) for admission to the hospital, a discriminating element to start the subject early to therapy with tocilizumab.

**Table 1:** Main comparisons between treatment groups (baseline clinical and laboratory features of IL group compared with CONTROL group).

	IL6 group (50 patients)	CONTROL group (62 patients)	*P*
Age, mean ± SD	61 ± 9	68 ± 14	.035
Gender, male (%)	70%	64%	.028
Hypertension (%)	34%	45%	.088
Diabetes (%)	6%	32%	.382
Charlson Comorbidity Index	2.08	2.9	.062
Antivirals^∗^ (%)	46%	56%	.161
Antimalarials^†^ (%)	100%	100%	
Glucocorticoids^‡^ (%)	100%	59%	.161
Antibiotics^§^ (%)	100%	100%	
LMWH (%)	42%	59%	.106
Lymphopenia (%)	98%	37%	.27
Non-invasive ventilation (CPAP) needed at admission	74%	37%	.205
Ferritin (medium value T0)	1592	1160	
d-Dimer (medium value T0)	2931	1087	

**Figure 1:**
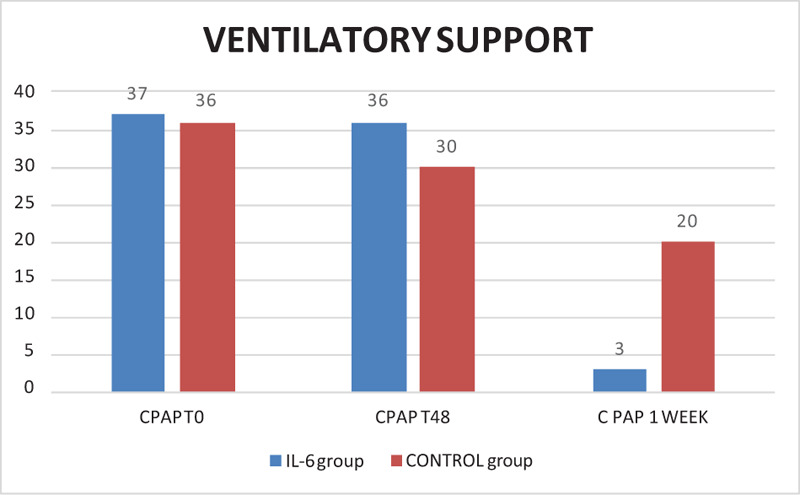
Ventilatory support needed before 48 h, after 48 h, and 1 wk after IL6-treatment (IL6 GROUP, blue column) and SOC therapy (CONTROL group, orange column) showing how patients of IL6 GROUP were weaned from O2 earlier than in the CONTROL group.

**Figure 2:**
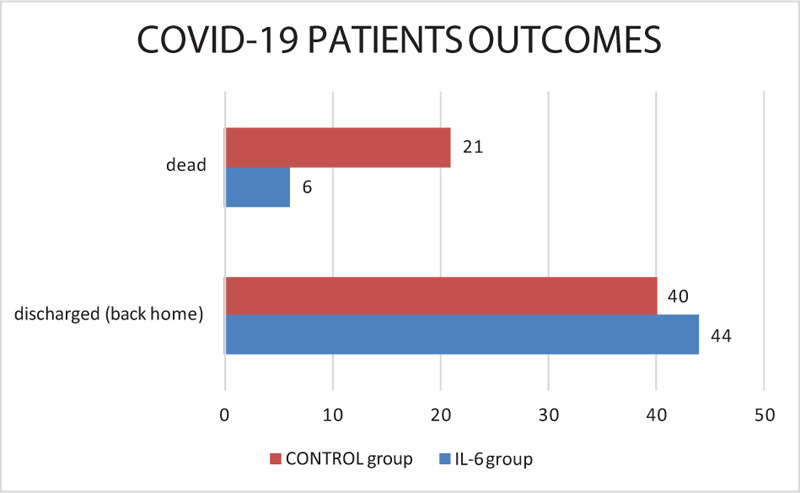
Summarized outcomes of treated patients, at time. IL6 GROUP outcomes are better than CONTROL GROUP, with a significantly higher number of patients discharged and a lower number of deceased patients.

Among IL6 group, 47 patients were treated with tocilizumab and 3 patients with sarilumab. Of them, 34% of the patients were hypertensive, 6% diabetic, and 5% had 2 or more comorbidity. At the onset of the COVID-19 symptoms, the majority had altered CPR, negative procalcitonin (PcT), mild lymphopenia (48/50 patients), and/or thrombocytopenia and hyperferritinemia. High d-dimer values (above 1500 ng/mL) were only sporadically documented and did not seem to be related to a more serious onset clinical picture. All patients received SOC according to SIMIT (Società Italiana Malattie Infettive) Diagnosis and Treatment Protocol.^[9]^ Anti-IL6 drug was administered on average 4 days after hospital admission, with a minimum of 1 day and a maximum expected time of 12 days. One patient received only 1 administration because of the appearance of skin rash. However, even in this patient, a good response to therapy was observed. Two patients received a third administration for worsening respiratory exchanges. In 2 patients, a rapid and marked radiographic improvement with a reduction in the interstitial thickening component was also observed. There were no adverse treatment events or infectious complications. In Figure 1, we summarized our final results. At the time of treatment, all patients needed oxygen support: 37 were ventilated with a CPAP mask (positive end-expiratory pressure [PEEP] 7.5–15 mm Hg), the remaining with a Venturi Mask (50%, 8 L/minute) and 2 patients with a 5 L/minute oxygen nasal flow. None of them were intubated prior to treatment. Most of them (46/50 patients, 92%) presented with fever, fatigue, dry cough, and dyspnea. We observed that body temperature and fatigue returned to normal on the first days after receiving IL6 treatment. The 3 patients treated with sarilumab improved slightly faster than those treated with tocilizumab. As illustrated in Figure 2, oxygen requirement in patients admitted from the second day after the monoclonal antibodies infusion decreased considerably. In the 72 to 96 hours following administration of the drug, the majority of patients were moved to a low-intensity area of care. Five patients needed transiently ventilatory support with CPAP only after IL6 drug infusion but none of them require non-invasive ventilation anymore. We observed a significant lowering in CRP value (*P* < .001). Ferritinemia, d-dimer, and PCT were not significantly different, instead. Between the variables collected, we observed a strong correlation between outcome (categorized as dismissed, improved, worsened, and deceased) and age of patients (Spearman's Rho 0.337, *P* = .018). Also, we observed a relationship between baseline CRP values and outcome (Spearman's Rho 0.284, *P* = .05). A subsequent logistic regression confirmed the link between age, baseline CRP and death with an ExpB/odd ratio of 1.22 and 1.16 respectively, *P* < .05.

After treatment, 5 patients required intensive ventilatory treatment and endotracheal intubation: 1 of them died, 2 are still intubated, and 2 are spontaneously breathing in air and dismissed. At the time of writing, 42 patients were discharged from the hospital, on average 17 days after IL6 infusion (range 7–27 days) and 20 days after their admission. Among the remaining patients, 2 are still hospitalized and intubated. To date, 6 of the 50 patients died. However, 5 of these subjects had a lot of comorbidities and a very severe disease prognosis. In these patients, the monoclonal antibodies were administered in the same way, considering the absence of valid therapeutic alternatives. Three of them died due to severe respiratory failure within a framework of severe acute respiratory distress syndrome (ARDS). One patient died of a serious cardiac myocardial infarction and 1 of massive pulmonary thromboembolism. In all 6 patients the treatment was proposed late, when the clinical conditions were already extremely serious and compromised.

Among the CONTROL group (40 M, 22 F, median age 68 years), 45% were hypertensive, 32% diabetic, and 16% had 2 or more comorbidity. At the time of treatment, all patients needed oxygen support and presented with fever, fatigue, dry cough, and dyspnea. At the admission, average values of ferritin, PCR, PcT, and d-dimer were absolutely superimposable on the IL6 group. On the date, all the 62 patients were discharged but 21/62 (33.8%) died (17/21 due to severe ARDS, 4/21 to bacterial sepsis).

## 4 Discussion and conclusions

SARS-CoV-2 infection is associated with a variety of pro-inflammatory mediators that may play important roles in the pathophysiology of the disease. Various drugs are being investigated for their potential utility in the pneumonia related to COVID. Improved understanding of molecular mechanisms regulating infection with this virus and the subsequent immune responses can potentially pave the way for any empirical approach, in the absence of proven effective treatments.

The SARS-CoV-2 infection may primarily affect T lymphocytes, particularly CD4+T and CD8+ T cells, resulting in a decrease in their numbers as well as IFN-γ production. These potential immunological markers may be of importance due to their correlation with disease severity in COVID-19. Numerous recent studies are showing that COVID-19 patients, express, as due patients affected by other coronaviruses, low levels of antiviral cytokines as IFN-gamma. Therefore, it has been speculated that this condition may promote Th2 responses with an up-regulation in the expression of Th2-derived cytokines such as IL4, IL13, and IL5 (5–7). In addition, a recent study has shown that patients requiring intensive care present a picture of perturbation of the cytokine structure with high cytokine levels, above all IL6.^[8]^ These alterations correspond to the cytokine release syndrome (CRS), which is an acute systemic syndrome characterized by fever and multi-organ failure associated with CAR-T chimeric antigen receptor CART cell therapy. The cytokines involved in its pathogenesis and clinical manifestations are IL6, interferon gamma (IFN-g), tumor necrosis factor alpha (TNF-a), and IL10. In particular, the central mediator in CRS toxicity is IL6.^[4]^ Increasing evidence is being documented on the potential key role of the anti-inflammatory therapy in the empirical treatment of emerging COVID-19 pneumonia.^[12]^ Although immuno-inflammatory therapy is not routinely recommended in COVID-19 pneumonia, in view of the CRS occurrence and the pulmonary anatomo-pathological findings of edema and hyaline membrane formation, a temporally targeted therapeutic approach accompanied by adequate ventilatory support could be beneficial in patients with severe pneumonia who develop ARDS. Thus, given the clinical condition and cytokine level in patients with severe COVID-19 pneumonia, tocilizumab may have a use in blocking virus-induced SIRS in patients with elevated IL6 levels. Based on the data collected, tocilizumab and sarilumab appear to be effective therapeutic alternatives for the treatment of medium to severe forms of pneumonia related to COVID-19, also in our, albeit limited, work. It seems that the occurrence of rapidly worsening systemic and respiratory manifestations (CPAP needing, respiratory rate increase, and/or rapid deterioration of oxygen requirements) and/or some biochemical markers of inflammation may represent a useful indicator for the identification of the ideal IL6-blocker responders. This may mean that early treatment with anti-IL6 drugs reduces the risk of mortality related to multi-organ failure, acting not only at the lung level but also at the systemic level, globally reducing the level of inflammation and therefore microvascular complications. Nevertheless, it is essential to identify the best window of opportunity for the treatment which, if delayed, is useless and self-defeating. After the treatment, the majority of patients treated had reduced inflammatory indices (CRP and ferritin); lymphopenia also progressively improved. We have not seen marked increases in liver function values. The resolution of the radiographic aspects seems to be slower.

Our data, although not supported by a randomized controlled trial, seem to suggest that the treatment with tocilizumab or sarilumab may be more effective in the early and rapidly progressive stages of respiratory distress, probably at the beginning of cytokine storm. Therefore, these drugs could be effective in an attempt to contain respiratory damage and delay or avoid mechanical ventilation.

Our data show that tocilizumab treatment must be started as early as possible to be effective: immediately in the case of patients presenting for observation with moderate to severe pulmonary insufficiency and need for non-invasive ventilation with CPAP or at the moment worsening of the respiratory picture or in the presence of hyperpyrexia not responsive to standard treatments. A treatment carried out in severely compromised patients is ineffective and burdened by numerous complications, fatal in most cases.

We knew that a limitation of this study is certainly the fact that all patients in the IL6 group were also treated with steroids while only a part of the control group received the same treatment; indeed, a preliminary analysis from a multicenter, randomized, open-label trial^[14]^ recently showed that patients randomized to receive dexamethasone had a lower mortality rate than those who received standard of care. Further studies and ongoing clinical trials certainly will help us to better define patients eligible as candidates for more aggressive intervention as well as response and therapeutic biomarkers. Despite the limitations of our series, it is possible that the use of monoclonal antibodies, when associated with standard-of-care therapy, can be useful in reducing the rate of intubated patients and respiratory complications. Furthermore, these treatments have the potential to reduce the time of hospitalization and, perhaps, the rate of overall mortality related to an emerging pathological condition such as COVID. Indeed, this disconcerting illness often occurs in extremely severe and rapidly progressive forms towards fatal frameworks and, to date, there are no codified nor highly effective therapies for this condition.

### 4.1 Uncited reference

^[7]^.

## Acknowledgments

We would like to thank all our colleagues and collaborators of the Medicine Unit of Legnano Hospital; Medicine Unit of Magenta Hospital; and Infectious Diseases of Legnano Hospital Alabardi Patrizia, MD; Baiardini Renata, MD; Bamberga Michele, MD; Biagiotti Sara, MD; Bompane Daniela, MD; Bonardi Giorgio, MD; Borsani Silvia, MD; Brivio Lorenza, MD; Capelli Francesca, MD; Caramma Ilaria, MD; Cardani Francesca, MD; Cimpanelli Maria Grazia, MD; Colombo Alessandra, MD; Conte Lucia, MD; Dalle Mule Ilaria, MD; De Rosa Stefania, MD; Diana Alessandro, MD; Evangelista Isabella, MD; Farina Francesca, MD; Ferrari Carlotta, MD; Grittini Alessandra, MD; Laria Antonella, MD; Lavazza Maria Teresa, MD; Magnani Carlo, MD; Marchesi Chiara, MD; Marchionni Lucia, MD; Mazzocchi Daniela, MD; Pavan Luca, MD; Porta Cesare, MD; Puricelli Simona, MD; Re Katia, MD; Speroni Sara, MD; Trolese Letizia, MD; Vacirca Valerio, MD; Venegoni Emanuela, MD for their valued contribution and support.

## Author contributions

CL designed and performed experiments, analyzed data, and co-wrote; LAM designed and performed the statistic analysis; TA contributed substantially to the conception and design of the study and the acquisition of data; MMG, OMCD, and RE contributed the acquisition of data; ZE and FP contributed the acquisition of data and their elaboration; MD and NM provided critical revision of the article; finally VPP and MA provided final approval of the version to publish.

**Investigation:** Maria Grazia Marrazza, Micol Olivetti, Daniele Colombo, Elisabetta Ricchiuti.

**Methodology:** Alfredo Lurati.

**Resources:** Eleonora Zaccara, Paolo Vigano’.

**Supervision:** Nicola Mumoli, Daniela Mastroiacovo.

**Visualization:** Faggioli Paola.

**Writing – original draft:** Antonio Tamburello.

**Writing – review & editing:** Laura Castelnovo, Antonino Mazzone.
